# Multi-Modality Imaging for the Identification of Arrhythmogenic Substrates Prior to Electrophysiology Studies

**DOI:** 10.3389/fcvm.2021.640087

**Published:** 2021-04-28

**Authors:** Alessia Gimelli, Sabine Ernst, Riccardo Liga

**Affiliations:** ^1^Fondazione Toscana Gabriele Monasterio, Pisa, Italy; ^2^NIHR Cardiovascular Biomedical Research Unit, Royal Brompton and Harefield NHS Foundation Trust, National Heart and Lung Institute, Imperial College, London, United Kingdom; ^3^Cardiothoracic and Vascular Department, Università di Pisa, Pisa, Italy

**Keywords:** electrophysiological procedures, ablation, atrial fibrillation, ventricular arrhythmias, imaging

## Abstract

Noninvasive cardiac imaging is crucial for the characterization of patients who are candidates for cardiac ablations, for both procedure planning and long-term management. Multimodality cardiac imaging can provide not only anatomical parameters but even more importantly functional information that may allow a better risk stratification of cardiac patients. Moreover, fusion of anatomical and functional data derived from noninvasive cardiac imaging with the results of endocavitary mapping may possibly allow a better identification of the ablation substrate and also avoid peri-procedural complications. As a result, imaging-guided electrophysiological procedures are associated with an improved outcome than traditional ablation procedures, with a consistently lower recurrence rate.

## Introduction

In the last decades, the role of noninvasive cardiac imaging has been greatly expanding, with all the available imaging modalities having now a relevant role in the characterization and risk stratification of patients with a wide range of cardiac pathologies. In this general context, the use of information obtained through noninvasive cardiac imaging has been steadily increasing also in the field of interventional cardiology, where an imaging-guided in-depth characterization of patients has become a mandatory step before any invasive procedure. Specifically, in the field of electrophysiology (EP), noninvasive cardiac imaging may be used to individuate the substrates of cardiac arrhythmias, selecting patients who will likely benefit from an ablation procedure (1–5). On the other hand, imaging is also used during the ablation to avoid complications and after the procedure to help in predicting arrhythmia recurrence. With these considerations in mind, we will aim at analyzing the role of noninvasive cardiac imaging before atrial and ventricular EP procedures, shedding some light on the possible future evolution of the field.

## Ventricular Arrhythmias

Considering the limited efficacy of pharmacologic therapies for the treatment of ventricular arrhythmias (VAs), cardiac ablation strategies have improved rapidly, becoming the reference techniques for the treatment of malignant VAs (1).

Similarly, noninvasive cardiac imaging has been also evolving, playing now a relevant role in the characterization of patients with VAs. For example, in patients with nonischemic cardiomyopathy (NICM), multimodality imaging can help in identifying the arrhythmogenic substrate and in characterizing the specific anatomical–functional components of the ventricular tachycardia (VT) circuits (2, 3, 6–9).

However, given the consistent etiological heterogeneity of the underlying myocardial disease (i.e., primary of acquired cardiomyopathy, inflammatory disease, and infiltrative pathology), the long-term success rate of cardiac ablation tends to be quite variable (10–12).

Patients with NICM are usually classified using their specific functional and structural phenotypic or genotypic findings (13).

Recently, Vaseghi et al. published a multicenter study on a population with all types of NICM submitted to VT ablation procedures. In the appraisal, a 69% 1-year success rate of catheter ablation was revealed, with better outcomes of patients with dilated cardiomyopathy, myocarditis, and arrhythmogenic right ventricular cardiomyopathy (ARVC) and a rather dismal prognosis of patients with hypertrophic cardiomyopathy (HCM), cardiac sarcoidosis, and valvular heart disease (14). The results of this study are very important, because they cover subpopulations of NICM that were not enrolled in previous published studies (10, 15, 16).

The different NICM etiologies exhibit different substrates of arrhythmogenicity, an aspect that is closely related to the efficacy of the ablation procedure. In particular, cardiac magnetic resonance (CMR) can be able to classify patients with ischemic cardiomyopathy (ICM) or NICM according to the presence of specific patterns of scar (17–19).

The pathophysiology of cardiac arrhythmogenicity in patients with ICM and a previous myocardial infarction (MI) is classically represented by an unexcitable necrotic area surrounded by a border zone of jeopardized myocardium with heterogeneous conduction that can predispose to the development of VAs (20).

Accordingly, considering the presence of a macroscopic arrhythmic substrate, it is not difficult to understand how ventricular ablation can be considered the treatment of choice in patients with VA in the setting of ICM, in addition or not to implantable cardioverter defibrillators (ICDs) (21).

Traditionally, VA ablation strategies were related to the identification of the re-entrant circuit, targeting its critical isthmus through activation map, using electro-anatomic mapping (EAM). In this case, despite a still sub-optimal success rate, catheter ablation significantly reduces disease-related hospitalizations and arrhythmic events when compared with conventional treatment (22). However, VA can be mapped in only 25% of the cases, because of hemodynamic instability or non-inducibility, and the recurrence of the arrhythmias after the procedure is in the range of 50% at 6-month follow-up (23).

The advent of contact mapping technologies involving multielectrode acquisition has represented a turning point for VA mapping, allowing a rapid delineation of the mechanisms of the arrhythmic circuit with higher resolution (24). Recently, Tung et al. demonstrated that myocardial reentry circuits are infrequently restricted to a single myocardial layer, involving the epicardium in 20–80% of patients (24).

In this regard, a recent paper by Soto-Iglesias et al. validated some imaging criteria for the identification of epicardial arrhythmogenic substrates in post-myocardial infarction patients with VAs undergoing CMR and cardiac computed tomography (CCT) (25). Accordingly, an epicardial scar area ≥ 14 cm^2^ in the late gadolinium enhancement (LGE) CMR images and a mean myocardial wall thickness on CCT ≤ 3.59 mm predicted the presence of the arrhythmogenic substrate as identified by EAM, showing the importance multimodality cardiac imaging for the characterization of scar-related VAs (25).

An alternative strategy is based on the technique of the ablation of the substrate that used the abnormal substrate evaluated during sinus or paced rhythm for targeting the areas with slow conduction (26, 27). In patients with ICM, this approach seems to have better results in terms of procedure success and outcome than standard electrophysiological mapping (28, 29).

One of the most promising substrate-based ablations is the ablation of local abnormal ventricular activities (LAVAs) (30), whose elimination through ablation seems associated with freedom from VT recurrence at long-term follow-up (31). Evidences accumulated in the last years have demonstrated that LAVAs are sharp high-frequency ventricular potentials (30) that represent near-field signals of slowly conducting tissue, which may represent VT isthmuses. Unfortunately, the accurate identification of the location of LAVAs depends on the specific mapping technique that is used.

Noninvasive imaging can be used routinely to identify different types of substrates, and it could be particularly helpful to define the location and extent of the VT substrate, in order to guide the pre-procedural planning.

### The ECG Evaluation

Even if the ECG is not considered by many as an “imaging” technique, it is always mandatory for the characterization of VAs and before every ablation procedure. A 12-lead ECG obtained during sinus rhythm is needed for the initial evaluation of the presence of underlying heart disease and is frequently able to identify the location of a myocardial scar (i.e., pathologic Q waves) as well as the possible origin of related VAs. In the presence of focal VAs that occur in the absence of structural heart disease (SHD), the ECG may indicate with a reasonable accuracy the site of origin of the arrhythmia. On the other hand, in the presence of VAs occurring in the setting of myocardial scarring (generally caused by reentry), the ECG abnormalities indicate the exit site from the reentrant circuit, which may not represent the best ablation target (1).

However, when the distance between the site of reentry circuit and the exit of the circuit is not so close, the ECG loses its impact in diagnosis, and other imaging techniques should be used to investigate better the focus' site (1, 5). Some algorithms have been proposed for the identification of the site of origin of VAs based on ECG analysis (32–34). Recently, Andreu et al. have validated an innovative ECG algorithm for the spatial identification of the myocardial segment of origin of the VT, which predicted accurately the results of EAM (82% same-segment agreement), independently of the underlying SHD and the site of origin of the arrhythmia (epicardial vs. endocardial) (34). Briefly, the algorithm combines the evaluation of the QRS axis in the frontal plane to locate the origin of the VA in the left ventricular (LV) short axis (inferior vs. anterior, septal vs. lateral) and the polarity in leads V3–V4 for its location in the longitudinal plane (basal, medial, or apical).

Accordingly, a 12-lead ECG should be performed in every patient with VAs and used to identify the region in which mapping efforts should be concentrated. However, more specific techniques should be used to pinpoint the actual site of ablation.

### Cardiac Magnetic Resonance

In recent years, a number of observational studies have shown how peri-procedural CMR imaging may allow targeting the arrhythmogenic substrate of VAs, possibly improving ablation success and ultimately reducing mortality (35, 36).

**Figure 1:**
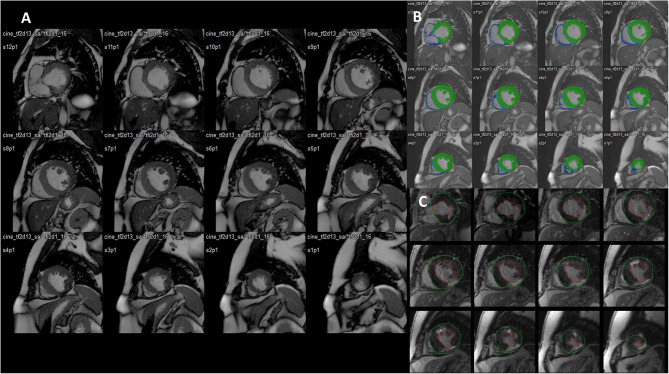
**(A)** Stack of cine images (end-diastolic frames) from a patient with previous myocardial infarction. Twelve parallel slices (top panels, basal segments; middle panels, mid-ventricular segments; bottom panels, apical segments). Slice thickness 8 mm, no gap. **(B)** The detection of the sub-epicardium and the sub-endocardium is performed semi-automatically (only the sub-epicardium is traced by the operator; mask mode). The left and right ventricle volumes and mass are automatically computed. **(C)** Late gadolinium enhancement images allow the precise depiction of two previous myocardial infarctions: one at the level of the inferolateral inferior wall (basal, mid, and apical segments), with a transmural involvement of the myocardium (transmurality: 75–100%), and one at the level of the anterior mid and distal segments with a non-transmural involvement (transmurality: 50%).

**Figure 2:**
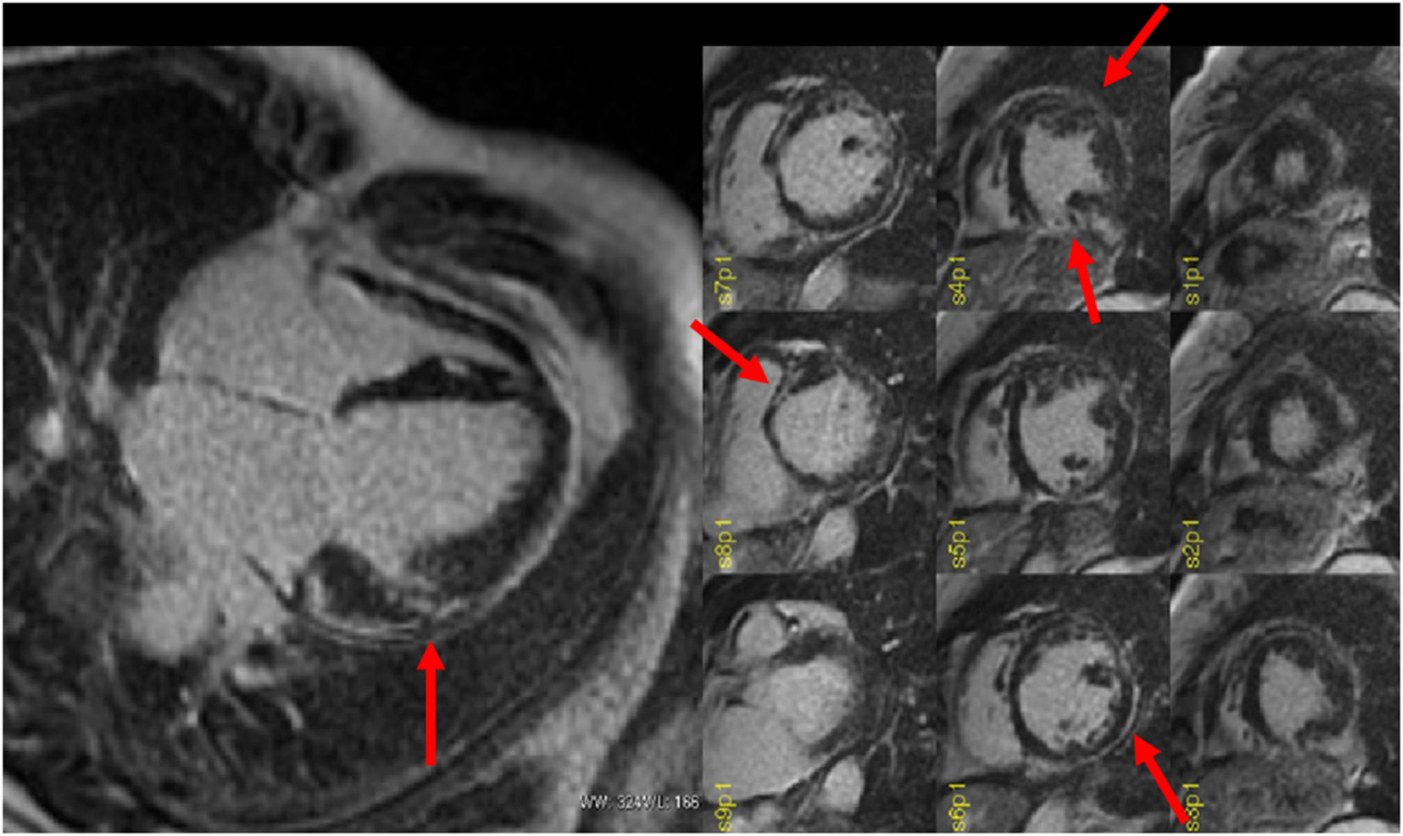
Late gadolinium enhancement (LGE) MRI images obtained in a patient with a previous history of myocarditis and with a recent history of hemodynamically stable sustained ventricular tachycardia (VT) scheduled for ablation procedure. The four-chamber (left) view shows the presence of a focal LGE area on the mid-ventricular segment of the antero-lateral wall (almost transmural). Short-axis images (right) show the real extent of LGE that also encompasses the inferior and lateral left ventricular (LV) walls (sub-epicardial), the antero-septal wall (midwall), and the distal anterior segment. Courtesy of Dr. Giovanni Donato Aquaro, Fondazione Toscana Gabriele Monasterio, Pisa, Italy.

In this setting, LGE imaging has been always considered the gold standard technique for the noninvasive delineation or myocardial scar (Figures 1, 2).

The area of necrosis identified by CMR correlates well with areas of low voltage revealed by EAM (6, 37–39). Hence, the three-dimensional (3D) integration of these two techniques seems instrumental for a better identification of the anatomical substrates on where to concentrate ablations (35, 36).

Most importantly, CMR imaging seems crucial when alternative access approaches are considered, such as in the case of epicardial ablations, where CMR may offer a complete assessment of the entire heart, including the epicardial myocardium and the pericardial sac.

Significant improvement has been made in the co-registration of CMR and EAM images, allowing accurate noninvasive navigation and planning of the ablation procedure.

The use of LGE images can allow identifying, by the quantitation of the intermediate signal intensity (SI), a mixture of fibrotic and viable tissue, called gray zone (40). The gray zone can be delineated using the areas with maximum SI and/or with remote myocardium (41). However, despite the evidence of the direct association between the extent of the gray zone and spontaneous and inducible arrhythmias, no consensus has been reached on its use to optimize the clinical treatment and the interventional procedure (41–45).

Classical studies have demonstrated the presence of pathologic myocardial fibrosis also in non-infarcted regions of patients with ICM, playing a possible role in arrhythmogenicity (46).

Interstitial fibrosis may be quantified with T1 mapping technique, associating with adverse prognosis (47). However, T1 mapping is not a direct measure of tissue dys-homogeneity and is usually confined to a limited number of preselected slices (48). In this respect, entropy has been proposed as a more direct measure of myocardial inhomogeneity derived from LGE images. It is computed from LGE images and quantifies the uncertainty of tissue composition as reflected by the uncertainty of SI (49, 50). Interestingly, when all the different CMR-derived parameters for the evaluation of myocardial structure were considered, scar-related entropy was associated with ventricular arrhythmogenicity, while the entropy of the entire LV is associated directly with mortality.

In the presence of right VAs, the use of LGE for assessment of arrhythmogenic site is limited by several factors. First of all, the right ventricle has a thinner wall than the LV, allowing the case for partial volume effect to occur (51, 52). Moreover, the peculiar motion of the right ventricle, particularly at the level of the outflow tract, may negatively impact LGE imaging. Accordingly, further studies and analyses should be performed before introducing this imaging technique in clinical routine.

**Figure 3:**
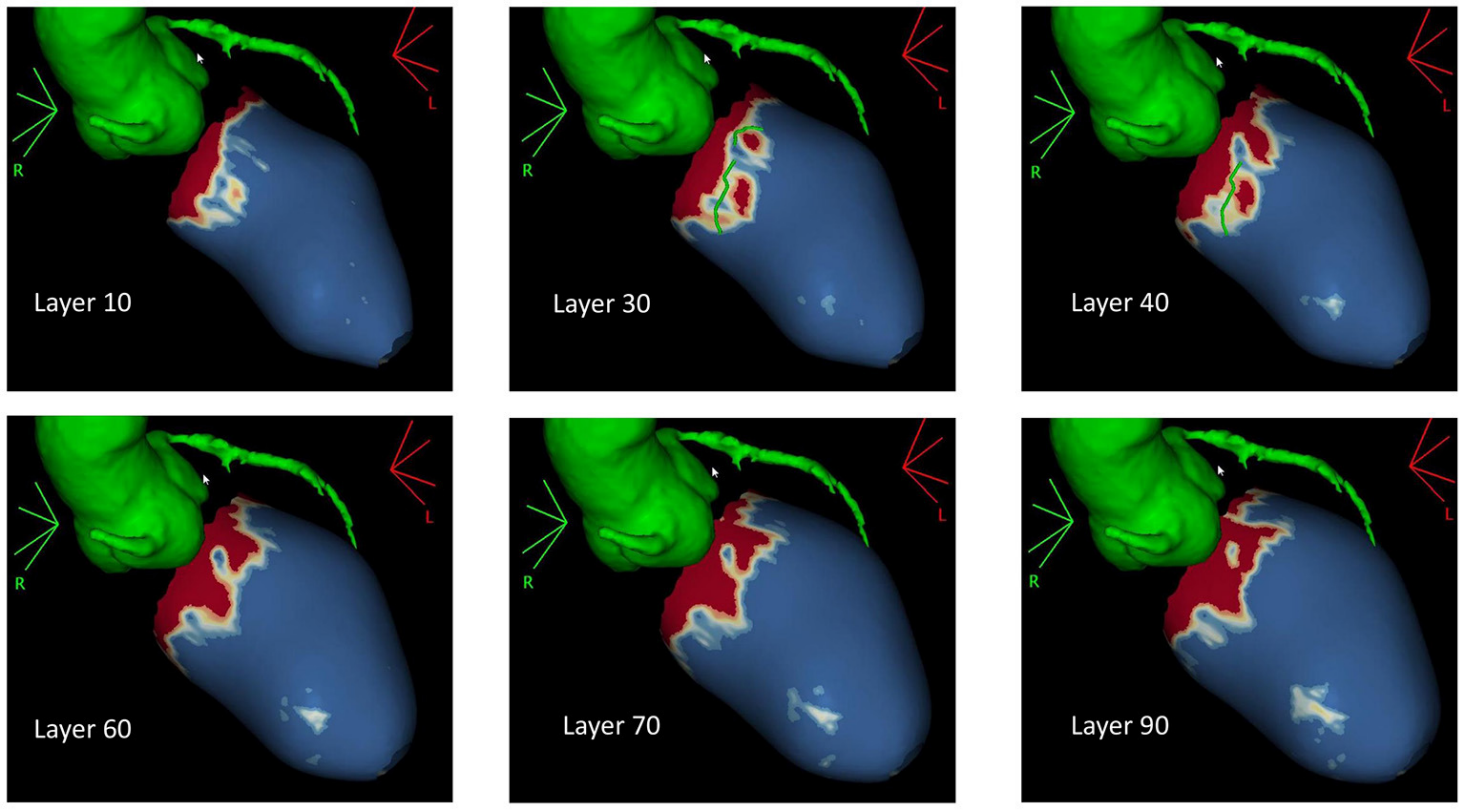
Pixel signal intensity maps obtained through post-processing of three-dimensional (3D) late gadolinium enhancement (LGE) MRI images at different layers of left ventricular (LV) wall thickness as obtained with ADAS3D software (ADAS3D Medical S.L., Barcelona, Spain). Conduction channels are evident on the mid-myocardial layers (30–40%) and are marked by green lines. Dense scar is depicted in red, while normal myocardium is represented in blue. Border zone tissue is shown in shades of red. Courtesy of Dr. Giulio Zucchelli, Second Division of Cardiovascular Diseases, Cardio-Thoracic and Vascular Department, University Hospital of Pisa, Pisa, IT, Italy.

In NICM patients waiting for VT ablation procedures, SI maps derived from LGE-CMR may be helpful to guide the procedure (35–53), because they allow a better detection of the arrhythmogenic substrate, and thus, they may reduce on the one hand the number of radiofrequency applications, and on the other, they can improve the efficacy of the treatment (54). Andreu et al. demonstrated that 3D LGE-CMR imaging had an added value in the pre-procedural identification of the anatomical conduction channels of a reentry circuit, defined as a region of border zone between two non-electrically conducting cardiac regions (i.e., two dense scars) (53, 54). Interestingly, 3D scar reconstruction using LGE imaging improved the identification of conduction channels prior to VT ablation (55). Specifically, pixel SI (PSI) maps obtained from LGE-MRI images allow the 3D endo-to-epicardial distribution of myocardial scar, localizing the presence of the conduction channels of the reentry circuit within the LV thickness (Figure 3) and possibly guiding the ablation procedure (4). Moreover, PSI maps derived from 3D LGE-MRI acquisitions may be conveniently fused with both CCT and EAM information, allowing the accurate navigation within cardiac structures (4, 56, 57). However, due to the still limited spatial resolution of MRI (1.4 to 2 mm for LGE images), the matching between the conduction channels identified with EAM and 3D scar reconstruction using LGE imaging is still imperfect, likely because of the inability of MRI to identify small bundles of myocytes, creating channels with limited conduction fibers that might still sustain the arrhythmia (55). Accordingly, despite the encouraging results, randomized studies are needed to confirm these findings.

Finally, LGE imaging may be crucial for the risk stratification of patients with NICM, allowing to identify those who would likely benefit from ICD implantation. In this respect, despite the results of the “Defibrillator Implantation in Patients with Nonischemic Systolic Heart Failure” (DANISH) study, showing a neutral impact on all-cause mortality of prophylactic ICD implantation in patients with symptomatic systolic heart failure (HF) not caused by coronary artery disease (CAD) (58), conclusive evidence has shown that the presence of LV scar on LGE imaging may identify those patients with NICM at risk of malignant VA that would benefit from an ICD (59, 60). In line with these evidences, already in the DANISH study, ICD implantation was associated with an almost 50% reduction of the rate of sudden cardiac death episodes, supporting the possible impact of ICD in a subset of patients with NICM (58). Similar results were reported also in patients with genetic dilated cardiomyopathies, further generalizing the prognostic role of LGE in patients with NICM (61).

### Cardiac Magnetic Resonance in Patients With Cardiac Implantable Electronic Devices

**Figure 4:**
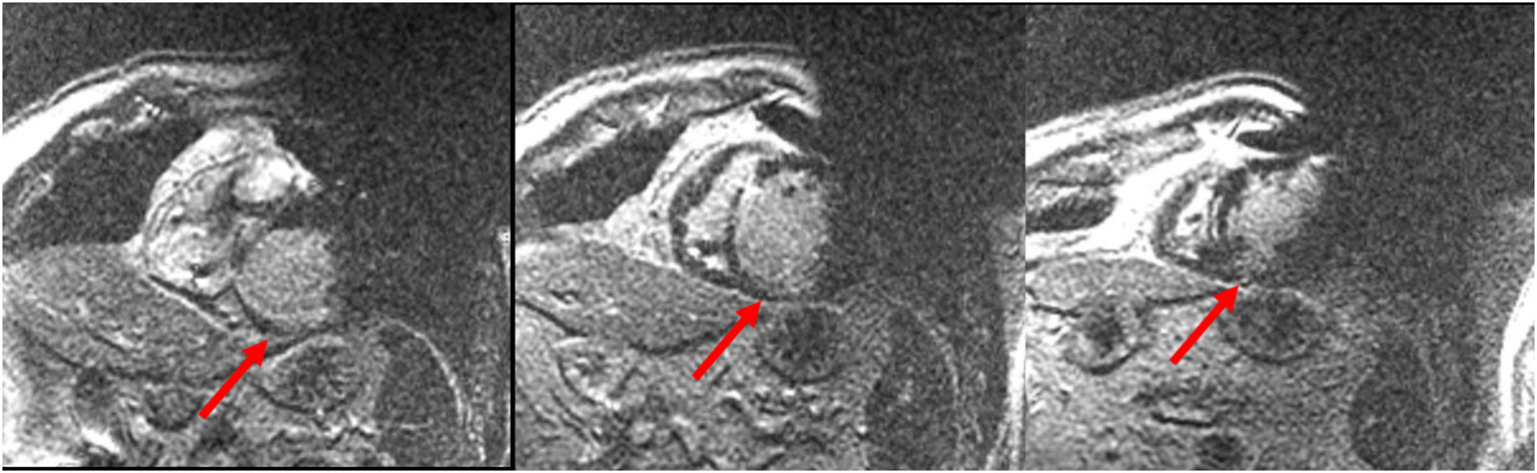
Cardiac magnetic resonance (CMR) images acquired with a wideband sequence in a patient with structural heart disease and an implantable cardioverter defibrillator (ICD) device. Image artifacts due to the ICD generator (right upper quadrant of the image) and coil (right ventricular cavity) are clearly visible. Nevertheless, a sub-endocardial inferior and infero-septal basal and mid-ventricular scar was revealed. Courtesy of Dr. Giulio Zucchelli, Second Division of Cardiovascular Diseases, Cardio-Thoracic and Vascular Department, University Hospital of Pisa, Pisa, Italy.

The presence of cardiac implantable electronic devices (CIEDs) has been generally considered a contraindication for CMR. However, several studies demonstrated that CMR could be performed safely in patients with CIEDs, if all the appropriate precautions are taken (62–64). It has been generally reported that metallic artifacts from the CIED could reduce the interpretability of myocardial signal, compromising the diagnostic power of the CMR study (65). However, recent improvements in the software algorithms have greatly reduced the degree of such artifacts, allowing a diagnostic CMR protocol in most of the patients with CIEDs. Specifically, in a recent study by Bhuva et al. a wideband LGE sequence has been shown to allow diagnostic LGE imaging in all the patients with left-sided CIEDs, including 79% of patients with ICD devices and nondiagnostic conventional LGE images (66). Examples of LGE images acquired in patients with CIEDs through a “wideband sequence” are reported in Figure 4.

### Cardiac Computed Tomography

Besides its known abilities in delineating coronary anatomy, CCT imaging may also allow an accurate identification of myocardial scar burden by delayed enhancement technique, which may individuate accurately the regions of low bipolar voltages on EAM (67). The integration of anatomical and functional data is possible in clinical routine, and the use of anatomical data provided by CCT imaging provides valuable information on the VT substrate that may conveniently integrate EAM (68).

The main advantage of CCT over CMR is the higher spatial resolution, which could reach the submillimeter range. Among the tissue characteristics that may indicate the presence of myocardial scar at CCT, a reduced wall thickness (<5 mm) (69), presence of hypoattenuation (70), and delayed contrast enhancement (67) are the most relevant. Interestingly, while CCT can be most valuable in patients with ICM, in patients with NICM, the accuracy of identifying scarring based on wall thickness alone is limited (68, 71), primarily because of the different nature and distribution of myocardial scarring, and hence arrhythmic substrate, in these conditions (67). As a result of that, the accuracy of CCT in identifying the arrhythmic substrate is lower in NICM than in ICM, possibly relating to limitations of the imaging technique in identifying areas focal scar intramyocardial scar (68, 71).

Finally, CCT allows the detection and quantitation of the epicardial fat thickness that is important in the pre-procedural ablation planning, because it can cover epicardial VT target sites (72, 73).

Moreover, the CT evaluation of epicardial fat can facilitate the correct interpretation of epicardial voltage maps, distinguishing epicardial fat from necrotic/scar area and increasing the efficacy of the epicardial energy applications. Finally, epicardial voltage readings may be used to identify the regions surrounded by fat, separating sites with and without overlying epicardial fat (72).

### Nuclear Imaging Techniques

Nuclear cardiac imaging has always represented the gold standard for the evaluation of myocardial perfusion, offering for a combined evaluation of myocardial blood flow regulation and tissue viability. However, the output of traditional perfusion nuclear imaging analysis is not sufficient to allow the precise identification of a VA focus, and, hence, perfusion imaging alone has a limited practical application in this setting (74, 75).

On the contrary, in the last decades, the possible clinical role of cardiac innervation imaging has been demonstrated, particularly in the context of patients at risk of VA. While different radiotracers are available for both positron emission tomography (PET) (76) and conventional [planar imaging and single-photon emission CT (SPECT)] nuclear imaging, ^123^I-metaiodobenzylguanidine (^123^I-MIBG) is generally used to this purpose (77). ^123^I-MIBG is generated by the combination of a benzyl group and the guanidine group of guanethidine, is characterized by high affinity for the pre-synaptic norepinephrine (NE) transporter (NET), and is similarly stored into vesicles as NE (77–79). Iodination of MIBG with a radioactive isotope enables successful imaging of sympathetic terminals. As a result, ^123^I-MIBG is internalized by pre-synaptic nerve endings of postganglionic neuronal cells through NET (77, 78). While planar imaging is generally used for the assessment of myocardial ^123^I-MIBG uptake, the additive value of SPECT has been more recently reported, providing information on regional myocardial sympathetic innervation (80).

Moreover, SPECT imaging may allow the combined evaluation of perfusion and innervation, in a regional as well as global way, enabling the localization and quantitative estimation of those jeopardized viable myocardial regions showing an impaired sympathetic innervation despite a still preserved perfusion (so-called “innervation/perfusion mismatch”) (81–83).

**Figure 5:**
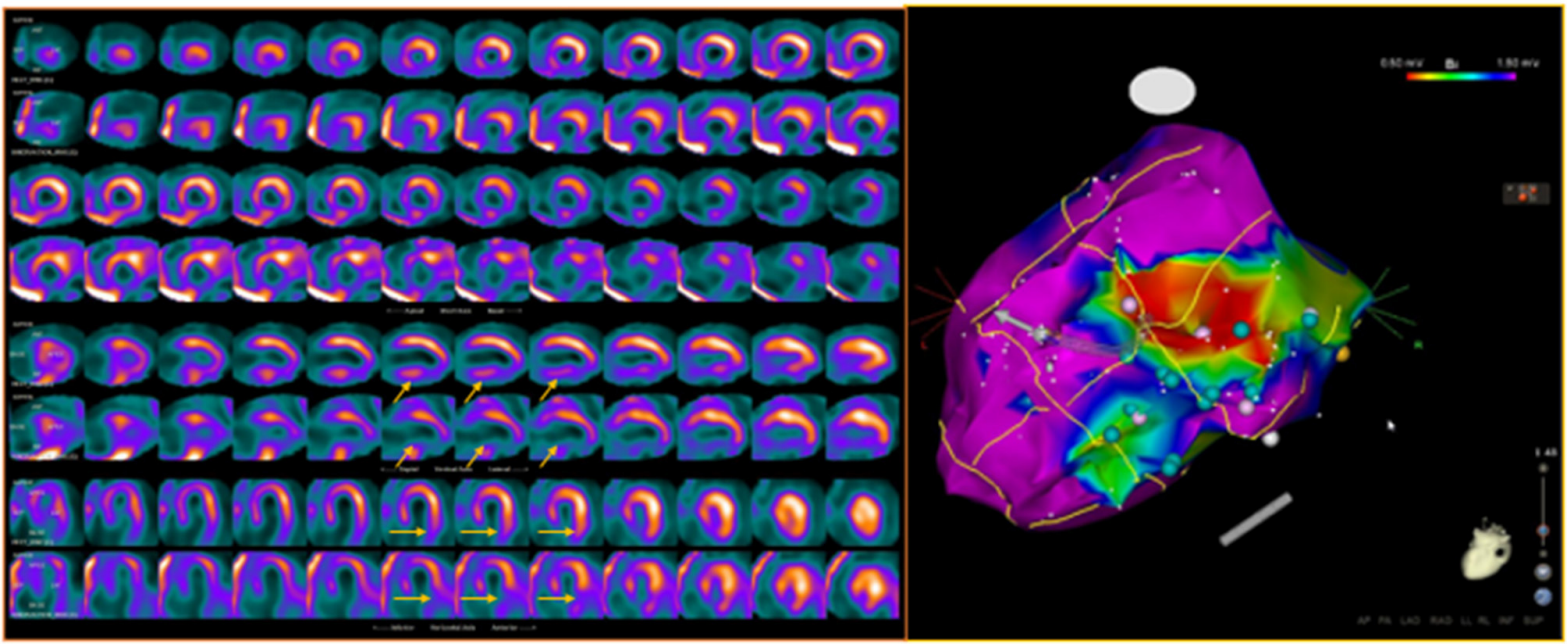
Dual-tracer scintigraphy in a patient with previous inferior myocardial infarction and recent episodes of ventricular tachycardia. Left: The ^99m^Tc-tetrofosmin rest single-photon emission CT (SPECT) images show the presence of an inferior myocardial infarction (top rows; SRS 12). The ^123I^-MIBG (bottom rows) innervation images showed a large area of absence of tracer uptake (no-innervation) from the basal to mid-ventricular lateral walls and in the inferior wall (SS-MIBG 19). A large area of innervation/perfusion mismatch was detected in the lateral left ventricular (LV) wall. Right: The electrophysiology (EP) study identified the arrhythmic focus in the inferior and infero-lateral LV walls, corresponding to the area of denervated myocardium. The ablation procedure was performed at the level of the inferior scar as well as of the viable infero-lateral wall. Pink dot, fragmented signals. White dot, late potential. Blue dot, double potential.

**Figure 6:**
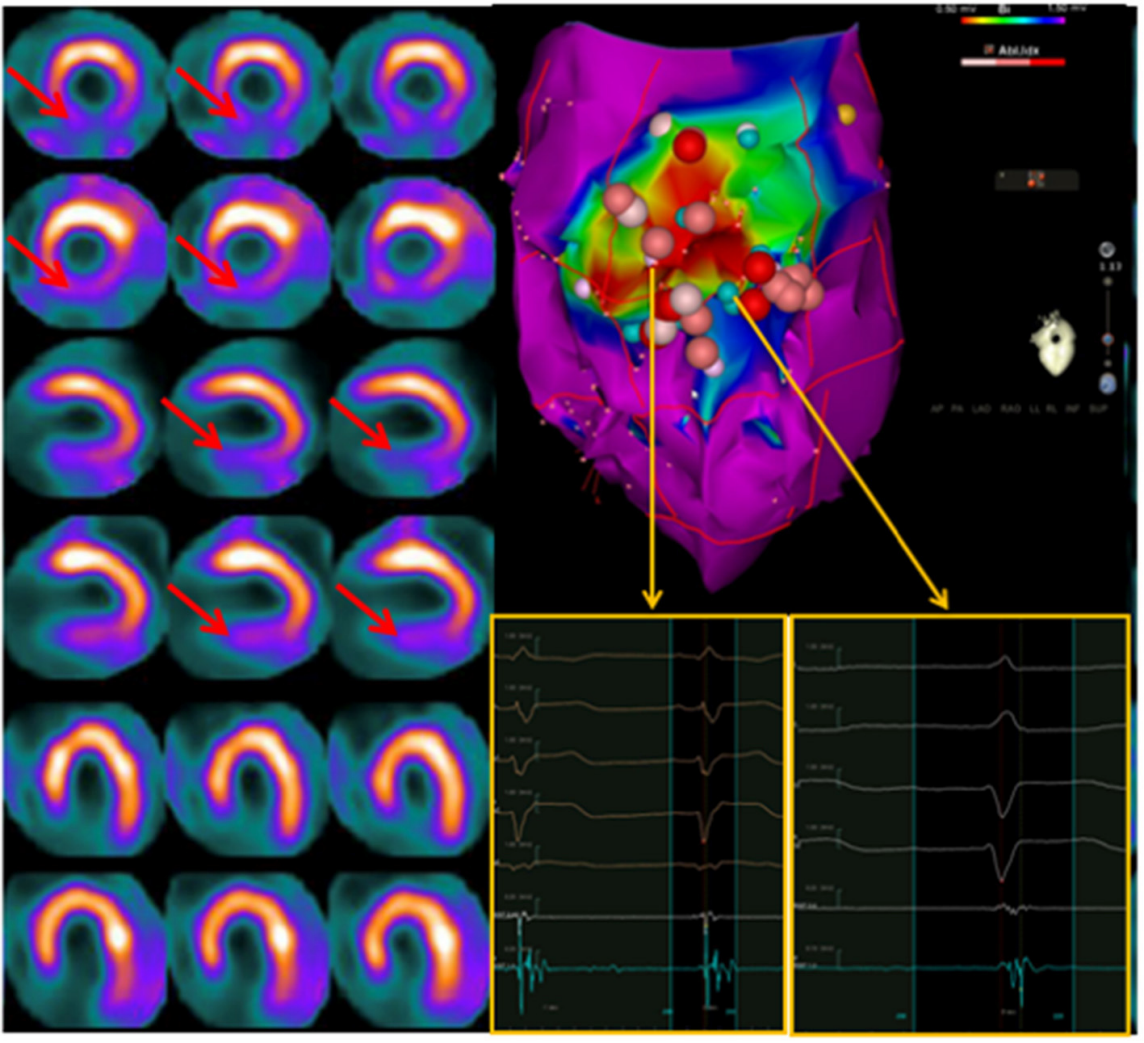
Left: Interaction between endocavitary ablations and regional myocardial innervation (^123^I-MIBG; top rows) and perfusion (^99m^Tc-tetrofosmin; bottom rows) parameters. Left: dual-tracer myocardial scintigraphy showing a basal inferior scar surrounded by a larger area of depressed sympathetic innervation (innervation/perfusion mismatch) in the mid-basal inferior and basal infero-lateral wall. Right: Left ventricle voltage map (inferior view) with correlation between perfusion/innervation mismatch and arrhythmic substrate, where the ablation was performed. On the side of the electrophysiological map are intracavitary signals of multichannel system (on light blue signals from ablation catheter). Adapted from Gimelli et al. (86).

In this respect, it has been repeatedly demonstrated that the location of areas showing an “innervation/perfusion mismatch” may co-localize with the sites of origin of the VA, representing possible ablation targets (84, 85) (Figure 5). In particular, in patients with ICM, the regions of “innervation/perfusion mismatch” are typically located at the level of the border zone of a scar, where the elevated concentration of NE (not re-uptaken by the dysfunctional sympathetic cells) can cause electrical instability (86) (Figure 6).

Accordingly, particularly in the setting of patients with ICM and VA, it is reasonable to foresee a wider application of combined innervation/perfusion nuclear imaging for the planning of complex ablation procedures and for the follow-up of patients.

More recently, PET imaging with ^18^F-fluorodeoxyglucose (FDG) has been proposed as an innovative noninvasive test for the noninvasive depiction of focal myocardial inflammation, which may represent a possible trigger of ventricular arrhythmogenicity. In this respect, PET imaging with FDG has been traditionally considered the reference standard for the noninvasive diagnosis of cardiac involvement of sarcoidosis (87). However, focal pathologic myocardial FDG uptake is not specific of CS, since it may rather indicate the presence of lymphocytic myocarditis in a significant proportion of patients (88, 89), a condition that, in the appropriate clinical context, may benefit from immunosuppressive therapy (90). In this respect, recent evidence has demonstrated how PET imaging with FDG may be conveniently used for the characterization of patients with VA, possibly identifying those with an underlying inflammatory cardiomyopathy and, hence, guiding patients' management. Specifically, as shown by Lakkireddy et al. approximately half of the patients with significant VA have underlying myocardial inflammation as demonstrated by focal FDG uptake, the majority of whom (58%) could be treated with just immunosuppressive therapy, thus avoiding ablation procedures (91).

### Echocardiography

While echocardiography maintains its role in the initial evaluation of arrhythmic patients, particularly in the setting of subjects with SHD, many would undermine its relevance in the setting of trans-catheter ablation procedures. In this context, echocardiography has been classically used to unmask or, better, rule out the presence of cardiac thrombi (92) prior to endocavitary ablation procedures. The use of contrast is particularly valuable in patients with inadequate ultrasonic windows and should be encouraged in order to increase the accuracy for the identification of thrombi (93). As a general rule, ablation procedures might be still performed in the presence of laminated thrombi (94), but in the absence of clinical urgency, a period of effective anticoagulation is strongly advisable. Intracardiac echography has gained increasing relevance in patients undergoing cardiac ablation procedures, primarily because of its unsurpassed spatial resolution in the definition of cardiac structures, and possibly increasing the success rate of the ablation procedures (95). Specifically, fusion of the anatomical reconstruction of cardiac chambers through intracardiac echography with EAM is generally performed to guide the ablation procedure and monitor possible adverse events (i.e., pericardial effusion).

Finally, in particular in patients with atrial fibrillation (AF), a preprocedural transesophageal echocardiogram should be performed to rule out the presence of left atrial (LA) thrombus and to minimize the risk of thromboembolic events, particularly when a transthoracic cardioversion is foreseeable (92).

## Atrial Fibrillation

AF is characterized by ineffective and uncoordinated atrial electrical activation and contraction. Despite being generally considered as a rather benign pathology, AF is associated with an increased risk of cardiovascular events such as stroke, heart failure, and death (96).

The physiopathology of AF is multifactorial and differs according to the type of disease (i.e., paroxysmal vs. persistent). As a general rule, the presence of a triggering mechanism as well as a substrate is believed to be necessary for the arrhythmia to become persistent (97). It has been classically demonstrated that in the majority of patients (particularly in those with the paroxysmal form of the arrhythmia), the triggers of AF are represented by arrhythmogenic zones mainly located around the ostia of the pulmonary veins (PVs) and less frequently around the superior vena cava or the coronary sinus. The basic invasive procedure for the treatment of AF includes the destruction of these triggers by isolation of the PVs and ablation of the other LA substrates.

Once the arrhythmia has developed, AF results in electric and structural atrial remodeling leading to progressive fibrosis, ultimately leading to the development of multiple reentry circuits that sustain the arrhythmia (98). Interestingly, besides atrial fibrosis, structural atrial remodeling involves also fatty infiltration, inflammatory infiltration, and amyloid deposition. In addition to structural atrial remodeling, electrical remodeling also takes place (i.e., by shortening the action potential duration), further sustaining arrhythmia (99). In addition to those well-known mechanisms, in the last decade, the additional role of impaired cardiac innervation in the pathogenesis of AF has been demonstrated. In fact, the atria are richly innervated by both the para-sympathetic and sympathetic nervous fibers, whose firing activity modulates both atrial mechanical and electrical functions (100).

Considering the complexity of the pathology, pre-procedural imaging may be helpful in patients with AF who are candidates for ablation procedures. Specifically, imaging allows to reconstruct the anatomy of the target cardiac structures anatomy and to anticipate future technical difficulties and risks of the procedure (101), possibly reducing ablation times and aiding in the management of complications.

### Echocardiography

Transthoracic echocardiography (TTE) is still the workhorse of noninvasive cardiac imaging, allowing to easily obtain key information on atrial structure and function. Accordingly, TTE is generally performed to evaluate LA size, a parameter that is used frequently to decide whether a patient will most likely benefit or not from an ablation procedure (5), being correlated with postablation AF recurrence (102). However, two-dimensional measurements of LA size tend to underestimate the true atrial volume on MRI or CT, making underrepresented the real number of patients with LA dilation (103). More recently, the use of speckle-tracking echocardiography allows the identification and the quantitation of atrial deformation (104) that may precede overt structural changes (105). However, speckle-tracking LA deformation analysis has not penetrated clinical routine also because of the lack of consensus on tracking methodologies (106).

In addition to that, 3D echocardiography may allow a finer evaluation of LA structure and function, entailing a complete assessment of the most relevant descriptors of chamber physiology, such as reservoir, suction, and pump functions (107). On the one hand, in patients with sinus rhythm, makers of LA anatomical and functional remodeling (i.e., larger LA minimal volume and lower LA ejection fraction) have been strictly associated with the development of AF (107). On the other hand, the same parameters may result in relevant predictors of AF recurrence after ablation procedure (108), outlining the relevance of and integrated evaluation of LA structure and function for the prognostication of AF patients.

### Cardiac Magnetic Resonance

CMR allows a multiparametric evaluation of atrial structure and function, with many MRI-derived parameters being correlated with postablation AF recurrence. Besides being able to quantify atrial volume in absolute terms, MRI allows the quantization of LA emptying function, as well as atrial strain and strain-rate whose abnormality may predict AF recurrence

Above all, MRI has been proposed as a valuable technique for the evaluation of atrial fibrosis, which has been recently classified into four stages: stage I (<10% fibrosis), stage II (10–20% fibrosis), stage III (20–30% fibrosis), and stage IV (>30% fibrosis) (109). In this respect, the DECAAF trial demonstrated that, in AF patients who submitted to catheter ablation, atrial fibrosis was independently associated with arrhythmia recurrence (110).

Interestingly, while the evaluation of LA fibrosis with LGE-CMR has generally remained controversial because of the limited reproducibility of the available methods, Benito EM et al. have been recently successful in standardizing the methodology for its evaluation, providing a reproducible tool for the identification of dense atrial fibrosis (111). Despite these results, the assessment of atrial fibrosis with CMR is limited to few specialized centers due to low availability and high specific training requirements (111–113).

The elevated spatial and temporal resolutions of cardiac MRI are instrumental for the in-depth characterization of LA function, allowing the estimation in measuring chamber relaxation, which has been repeatedly associated with AF development (114, 115). In particular, the LA stiffness index, as an integrated parameter of LA diastolic function obtained from a combination of noninvasive volume measurements from MRI and invasive pressure measurements at the time of the LA ablation, was higher in patients with persistent AF and double in those with recurrence of AF after LA ablation (115).

### Cardiac Computed Tomography

CCT has an invaluable spatial resolution that makes it ideal for the pre-procedural cardiac anatomical assessment. CCT measurement of atrial volume correlates well with CMR (116). Moreover, innovative parameters have been recently proposed for the characterization of patients with AF. Among others, LA sphericity index, as an innovative index of atrial remodeling, has been recently shown to further stratify patients' risk for arrhythmia recurrence following ablation (117). CCT can provide an accurate anatomical reconstruction of the LA, depicting PV anatomy and unmasking possible anatomical variants, all representing key data for procedural planning.

**Figure 7:**
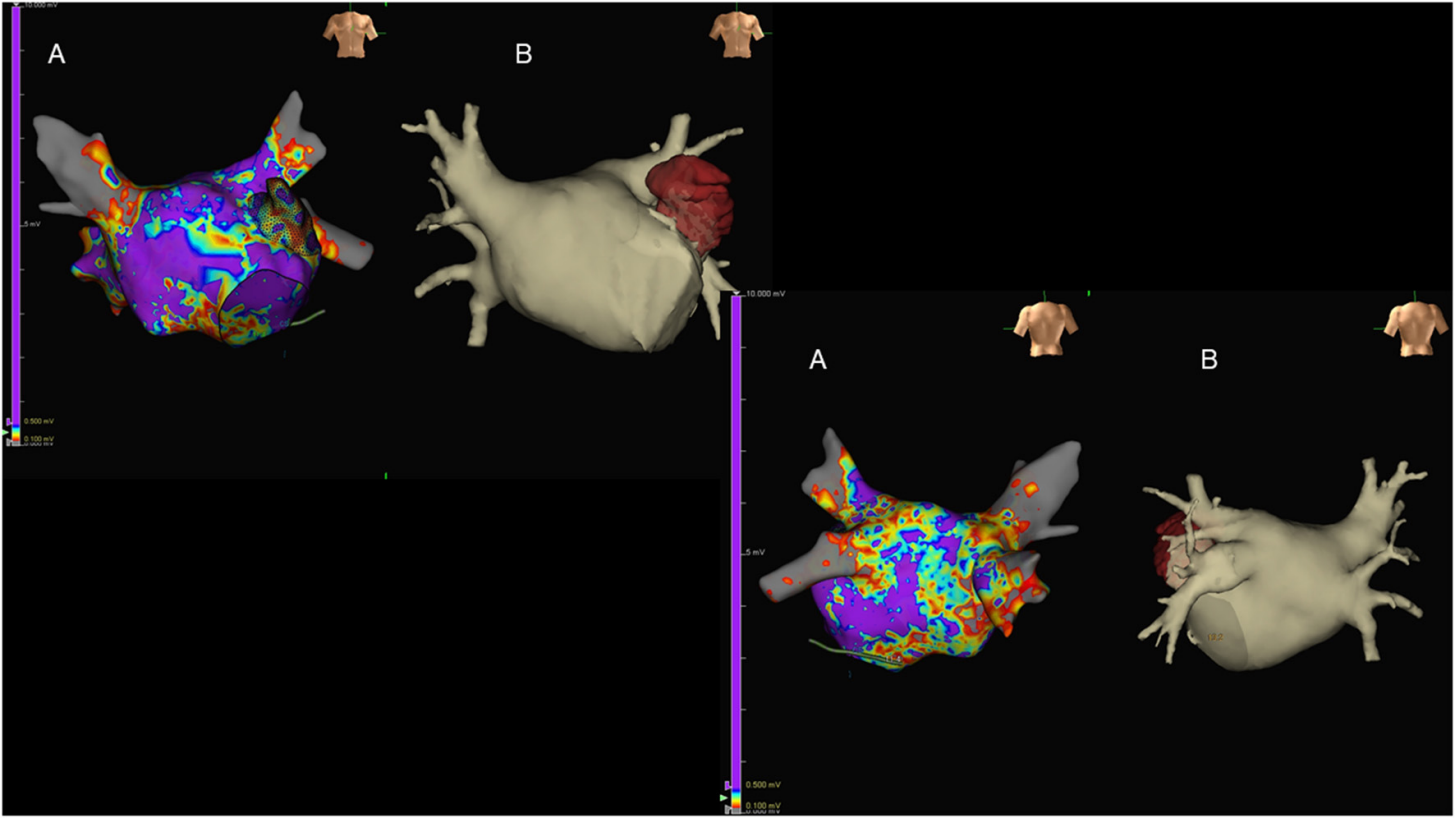
Antero-posterior (upper-left images) and postero-anterior (lower-right images) views of the left atrium (LA) in a patient with long-lasting atrial fibrillation submitted to endocavitary ablation. Three-dimensional (3D) LA anatomy was built by means of EAM [**(A)**; Ensite Precision, St. Jude Medical, USA], aided by volume-rendered CT coronary angiography (CTCA) images **(B)**. Of note, LA voltage maps were superimposed on the reconstructed 3D LA volume, showing widespread LA disease with diffusely reduced voltages evident.

CCT images are generally fused with EAM, allowing the match between anatomy and electrical signals and improving procedural efficacy (118). If compared with CMR, a CCT acquisition is characterized by a significantly lower time for both acquisition and data analysis, at the cost of a non-negligible radiation exposure. Accordingly, the choice between modalities depends on local availability and patient specific characteristics. An example of the integration of anatomical information derived CCT with functional data obtained through EAM in a patient with AF is reported in Figure 7.

### Nuclear Cardiology

As previously demonstrated, there is a strong correlation between AF and abnormal activity of the cardiac autonomic nervous system (119). Cardiac ^123^I-MIBG scintigraphy allows the noninvasive evaluation of myocardial sympathetic tone. After the first episode of paroxysmal AF, a reduced late H/M may predict the development of permanent AF at follow-up (120), and a high ^123^I-MIBG washout may in turn predict AF relapses in both the paroxysmal and permanent AF settings (121). SPECT imaging may allow a finer characterization of the spatial distribution of MIBG, providing more accurate measures of sympathetic dysfunction. Accordingly, when SPECT MIBG imaging was performed in patients with AF undergoing ablation, the presence of regional innervation defects after the procedure was associated with an increased risk of AF relapses at short-term follow-up (40% vs. 17% of patients) (122).

**Figure 8:**
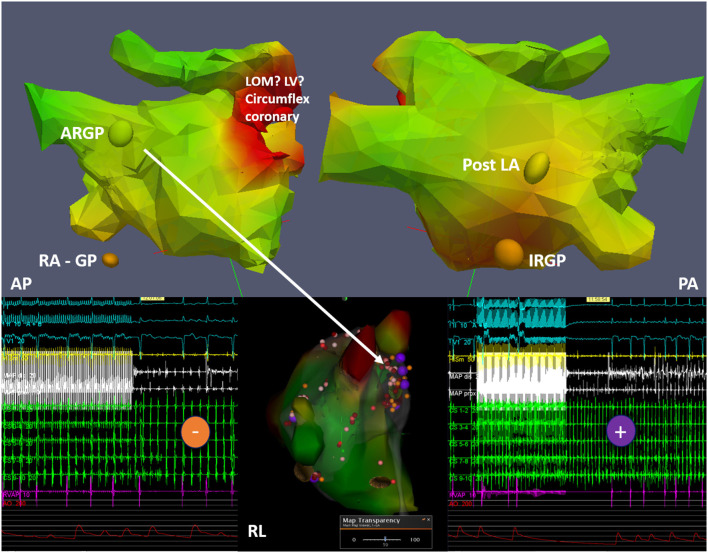
Example of ^123I^-MIBG imaged-guided ganglionated plexus (GP) modulation in a patient with paroxysmal atrial fibrillation. The top panels depict three-dimensional (3D) reconstructions from ^123I^-MIBG nuclear image merged with contrast computed tomography imaging with discrete uptake sites of ^123I^-MIBG demonstrated as colored spheres (ARGP, anterior right GP; IRGP, inferior right GP; RA GP, right atrial GP; post LA GP, located in the posterior LA wall). The middle lower panel shows the integration into the electroanatomical mapping system with the results of the high-frequency stimulation (HFS) shown as colored tags (purple, positive HFS effect with temporary AV block; orange, negative HFS effect). In addition to radiofrequency ablation at positive HFS sites, conventional isolation of the pulmonary veins is subsequently performed. Other abbreviations: AP, antero-posterior projection; PA, postero-anterior projection; RL, right lateral projection; LV, left ventricle; LOM, ligament of Marshall.

More recently, the role of intrinsic atrial innervation dysfunction on the pathogenesis of AF has been explored. In fact, the cardiac autonomic system includes neurons located in the ganglionated plexi (GPs) in the epicardial fat pads that overlie the atria (123). Four of the seven main GPs are located around the PVs, and the long-term results of the ablation procedure may depend on the effective destruction of these GPs (124). The standard approach to localize the GPs is to apply high-frequency stimulation (HFS) to the presumed GP areas, but besides being invasive in nature, this method has low accuracy (124). ^123^I-MIBG imaging has been used to localize GPs. However, considering the average size of GPs (5–10 mm), the spatial resolution of traditional SPECT cameras may be inadequate to this purpose. The novel cardiac cameras equipped with cadmium–zinc–telluride (CZT) detectors offer a significantly increased resolution than standard Anger cameras, appearing more suited for the localization of GPs. Recently, Stirrup et al. defined a high-resolution CZT SPECT/CT protocol to identify GPs with good accuracy and reproducibility (125) (Figure 8). If confirmed by further studies, LA innervation imaging by SPECT might refine the planning of the ablation procedure and help to predict AF recurrences.

#### The Role of Cardiac Imaging in Patients' Risk Selection

Despite the consistent improvement of catheter ablation techniques, a consistent proportion of patients with either atrial or VAs still relapse after the procedure. In fact, in the recently completed “Catheter Ablation vs. Antiarrhythmic Drug Therapy for Atrial Fibrillation” (CABANA) trial, ~50% of the patients randomized to catheter ablation relapsed during 48.5 months' follow-up (126), a figure also confirmed by the long-term results of the “Catheter Ablation vs. Standard Conventional Therapy in Patients with Left Ventricular Dysfunction and Atrial Fibrillation” (CASTLE-AF) trial in population of patients with a much higher cardiovascular risk (127). Accordingly, those data point to the need of a better selection of the patients who are submitted to AF ablation in order to avoid futile interventional procedures.

Similarly, according to recent appraisals, the recurrence rate of VT ablation procedures is far from being excellent, with a still significant proportion of patients with SHDs relapsing at short-term follow-up. In particular, 50% of patients with sarcoidosis and 43% of those with valvular cardiomyopathy submitted to VT ablation recur after 1 year (14). On the contrary, VT recurrence if significantly less frequent in patients with dilated (32% at 1 year) cardiomyopathy (14), pointing to the need of a finer patient characterization before proceeding to complex ablation procedures.

In this context, multimodality cardiac imaging may play a pivotal role in pre-procedural patients' characterization and risk stratification, possibly allowing the selection of only those patients who would more likely benefit from an invasive approach (Table 1). For example, the demonstration of severe atrial remodeling on 3D-echocardiography or MRI may identify patients in whom a conservative approach could be first attempted (107, 108). Similarly, the evidence of a complex cardiomyopathy, possibly with extensive LV scar burden on nuclear imaging or MRI, would reasonably discourage an interventional approach (14, 128).

**Table 1:** 3D image integration options for electrophysiology.

**Modality**	**Added information**	**Comment**
Intracardiac echocardiography	Allows 3D reconstruction, directly integrated in EAM systems, real-time assessment	Not available everywhere due to high costs requires large bore (9 Fr) access.
Cardiac computed tomography	3D roadmap reconstruction from DICOM data for most 3D EAM systems	Images only cardiac chambers that are contrast filled (difficult in congenital heart disease where contrast transit times may be very delayed). Potentially nephrotoxic contrast agents. Narrow imaging window may not allow imaging of more peripheral vessels.
Non-contrast MRI	3D roadmap reconstruction from DICOM data is feasible from whole blood sequence	Shows all chambers that are blood-filled, and a large field of view can be applied to assess access pathways. Subject to artifacts from implanted devices.
Late iodine enhancement CT	Allows scar imaging and 3D reconstruction. Software for 3D image integration available (several commercial providers available)	High contrast burden and radiation exposure, but no problem with artifacts from implanted devices.
Late gadolinium enhancement MRI	Allows scar imaging and 3D reconstruction. Software for 3D image integration available (several commercial providers available)	Excellent scar imaging for ventricular scar; limited data for atrial scar so far. Warning for gadolinium in severe renal dysfunction.
Conventional nuclear imaging (SPECT)	High-resolution MIBG ± perfusion imaging can be fused with CT/MRI data	Experimental evidence so far for atrial fibrillation and ventricular tachycardia ablation.
PET-CT	Metabolic or perfusion markers available	Experimental evidence; no 3D image integration yet commercially available.

*3D, three-dimensional; EAM, electro-anatomic mapping; SPECT, single-photon emission CT*.

## Conclusions

Cross-sectional imaging can be used to integrate information and to plan different kinds of electrophysiological procedures, thanks to the evaluation of anatomy, functional abnormalities, and the substrate. Furthermore, noninvasive imaging can optimize the procedure by fusion with the mapping system.

Finally, image-guided electrophysiological procedures were found to have better short- and long-term outcomes than were conventional procedures.

## Author Contributions

AG, SE, and RL wrote the manuscript and prepared the figures. All authors contributed to the article and approved the submitted version.

## Conflict of Interest

SE declares the following: consultant for Biosense Webster, Stereotaxis Inc, and Spectrum Dynamics; and grants from Catheter Precision, Spectrum Dynamics, and Baylis Medical. The remaining authors declare that the research was conducted in the absence of any commercial or financial relationships that could be construed as a potential conflict of interest.
